# The Use and Benefits of Focused Shockwaves for the Diagnosis of Myofascial Pain Syndrome by Examining Myofascial Trigger Points in Low Back Pain

**DOI:** 10.3390/biomedicines12122909

**Published:** 2024-12-20

**Authors:** Hannes Müller-Ehrenberg, Federico Giordani, Alessandra Müller-Ehrenberg, Richard Stange

**Affiliations:** 1 Private Clinic Orthopädische Privatpraxis, 48143 Münster, Germany; info@triggerpunktzentrum.de; 2 Villa Rosa Rehabilitation Hospital, APSS Trento, 38122 Trento, Italy; 3 Faculty of Health, Witten/Herdecke University, 58455 Witten, Germany; alessandra.mueller-ehrenberg@uni-wh.de; 4 Department of Regenerative Musculoskeletal Medicine, Institute of Musculoskeletal Medicine, University Münster, 48149 Münster, Germany; richard.stange@ukmuenster.de

**Keywords:** Extracorporeal Shockwave Therapy (ESWT), low back pain, myofascial pain syndrome, myofascial trigger points, pain, diagnosis

## Abstract

**Background/Objectives**: Low back pain (LBP) is a widespread public health issue, with myofascial pain syndrome (MPS) being a common cause, affecting 67–100% of patients. However, there are significant challenges in the diagnostic process due to the subjective and unreliable nature of manual palpation. Focused Extracorporeal Shockwave Therapy (F-ESWT), traditionally used for MPS treatment, offers a reproducible and non-invasive mechanical stimulus, making it a potential diagnostic tool. This study evaluated F-ESWT’s diagnostic efficiency in chronic LBP patients by focusing on “recognition” and “referral” of pain. **Methods**: twenty-eight participants were screened for myofascial trigger points (MTrPs) in the lumbar, gluteal, and thigh regions. Identified MTrPs were stimulated using F-ESWT, and patient feedback was recorded. **Results**: data showed high diagnostic accuracy for muscles such as the quadratus lumborum, gluteus medius, and gluteus minimus muscles, achieving “referral” rates of 96%, 95%, and 92% and “recognition” rates of 84%, 86%, and 85%, respectively. Other structures like adductors, iliopsoas, erector spinae, and biceps femoris muscle showed consistent but lower diagnostic rates. **Conclusions**: the study’s findings indicate that F-ESWT effectively reproduces pain patterns, offering a precise, reproducible, and non-invasive diagnostic approach for MPS in chronic LBP. However, they also highlight the necessity for detailed diagnostic criteria in managing myofascial pain.

## 1. Introduction

For more than two decades, Extracorporeal Shockwaves Therapy (ESWT) has been established as a standard procedure for the treatment of musculoskeletal disorders due to its ability to reduce pain and accelerate regenerative processes in the treated tissue (International Society for Medical Shockwave Therapy, ISMST Guidelines, www.ismst.com). In recent years, there has been a growing emphasis on the use of ESWT for the treatment of myofascial pain [1,2,3,4]. Myofascial Pain Syndrome (MPS) is a clinical condition and one of the most common causes of pain [5]. The established authors describing pain originating from myofascial tissue emphasize that the presence of at least one myofascial trigger point (MTrP) is the central element in the diagnosis of MPS [6,7]. MTrPs are hyperirritable points within a tight band of skeletal muscle that manifest with local and referred pain, decreased range of motion, and often autonomic phenomena [8,9,10,11]. Diagnostic criteria have been established to clarify the diagnosis of MPS and allow differentiation from other soft tissue disorders [7,12,13]. Diagnostic Criteria, according to Travell/Simons, are (1) Taut band in myofascial tissue, (2) Tenderness/Palpaple knot (MTrP) on the taut band, (3) Referral of pain and (4) Recognition of pain (5) Local Twitch Response. The criteria most commonly used by researchers and expert clinicians include all those previously mentioned by Travell and Simons, with the exception of the local twitch response, which has not yet been fully validated as a reliable diagnostic test [7,14,15,16,17,18,19]. In addition to specific examination and palpation, clinicians should inquire about the location, frequency, and characteristics of the pain while stimulating a MTrP. They should ask, “Is this pain part of your usual complaints?” and “Does the pain refer anywhere from the spot that I am compressing?” [5,6,20]. A positive response to the first question indicates the ability to recognize pain, while a positive response to the second question indicates the presence of referred pain [5,6,12,20].

The use of diagnostic criteria helps to classify the patient’s complaints into a reproducible diagnostic system [18,21]. In general, the reproduction of pain and the patient’s feedback are an important part of any clinical examination and are useful for understanding the patient’s complaints. There is a consensus that the most important criterion in MPS is “recognition”, whereby the pain elicited by stimulation, usually palpation, of the trigger point is recognized by the patients as their “familiar pain” [13,14,18,22,23]. Recognition of the complaints by precise stimulation may indicate that the stimulated structures are involved in the whole process or even dominantly cause it [8,13,14,24]. Another important diagnostic criterion and characteristic feature of myofascial pain is “referral of pain” [8,9,13,25]. This means that the pain is not only local but radiates into more distant parts of the body. A typical example is the pseudo-radicular radiation in the lower extremity from the gluteal muscles, as described in the textbooks [6,20,26].

The standard for the diagnosis of MTrPs is still physical examination by palpation, which suffers from issues of subjectivity and inter-rater variability [7,12,14,15,16,17,19,21,27]. In the scientific context, the available clinical tests and manual examination techniques for LBP are reported to be insufficiently accurate [28]. In the specific case of examination for myofascial pain, physical examination with palpation is considered by some authors to be unreliable in adequately diagnosing MTrPs [14,15,19,29,30]. In addition, both inadequate use and a lack of transparency in the reporting of MTrP diagnostic criteria are noted in the literature [30,31]. While other diagnostic modalities such as intramuscular needling, surface electromyography, infrared thermography, elastography, and ultrasound have been developed, they have not yet reached the standard of clinical use and scientific acceptance [14,32,33,34].

Therefore, there is a need for further improvement of MTrP diagnostics to provide more robust data for MPS as a source of pain. Thus, new tools capable of standardizing and reproducibly identifying MTrPs are needed for clinical practice [14,35]. Among many, F-ESWT is a non-invasive tool that allows rapid access to myofascial structures [36,37]. In recent years, a few physicians stated that when F-ESWT is applied to MTrPs, the precise stimulus of the focused shock wave established the diagnostic criteria well, also from deeper tissue layers [38,39,40]. In clinical use, the patient’s feedback confirms the exact application of F-ESWT on MTrPs [38,39,40,41,42,43,44].

Low back pain (LBP) represents a significant public health concern in contemporary society. It is estimated that the lifetime prevalence of LBP is between 30% and 80%, depending on the population and increases with age [45,46,47]. A diagnosis of “specific LBP” is made on the basis of scientifically approved examinations and indicates that the pain is attributable to conditions such as spondylitis, trauma, fractures, tumors, infections, vascular, metabolic, or endocrine-related processes [48]. In the majority of cases of LBP (over 80%), the causes could not be verified using the scientifically recognized examination methods employed to date and are therefore labeled as non-specific LBP [28,48,49,50]. Nevertheless, muscles and fascia are cited as one of the main causes of LBP in a large number of publications [12,13,21,22,23,24,29,35,51,52,53,54,55,56,57,58,59].

MPS in LBP has a high prevalence ranging between 67% and 100% [12,22,29,52]. Therefore, MPS, and myofascial trigger points (MTrPs) in particular, have become increasingly important among experts in the management of LBP [12,22,29,52,60,61]. Following the use of ESWT for the treatment of myofascial structures [3,38,39,40,42,43,62], studies have been published using these structures as targets for the treatment of LBP [44,51,63,64,65,66]. Despite the absence of a widely accepted therapeutic protocol for myofascial shockwave therapy, the results of previous studies indicate that ESWT yields comparable or even superior outcomes to other treatment modalities (e.g., TrP-injection, etc.) [1,3,38,41,64,67]. It is noteworthy that the majority of studies do not adhere to a treatment protocol aligned with the principles of myofascial pain diagnosis and therapy [1,2,3,4]. In many clinical studies, the exact area of shockwave application is not described [1,2,3,4,51,63]. A limited number of previous studies have documented the mode of application, the practitioner’s experience in diagnosing and treating myofascial pain and myofascial trigger points, and the use of ESWT [1,2,3,4].

**Figure 1:**
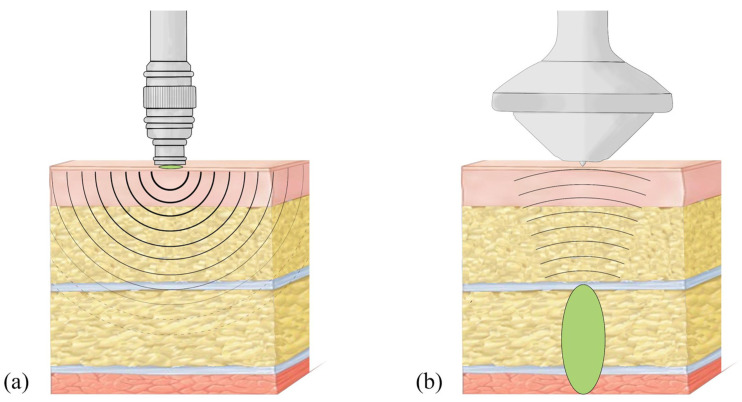
Schematic representation of (**a**) radial pressure wave (R-PP) and (**b**) focused shockwaves (F-ESWT) applied on a section of the soft tissues (from top to bottom: skin, adipose tissue and fascia, muscles). Focused zone (green) and wave propagation (black lines) are shown for both technologies. Copyright by Federico Giordani.

In addition, two principal forms of ESWT have been employed thus far: focused shock wave (F-ESWT) and radial pressure pulse (R-PP). It is erroneous to designate R-PP as a radial shock wave, as the physical characteristics diverge significantly. Thus, a clear distinction between radial and focused shockwaves is essential to assess its diagnostic and therapeutic potential. The medical devices of both energy forms deliver a reproducible, standardized mechanical stimulus to the tissue. F-ESWT generates acoustic pressure waves that are bundled and converge at a certain tissue depth (the focal zone) in the human body, reaching even deeper tissue layers in a non-invasive, targeted manner [68]. In contrast, the radial pressure wave achieves a significantly lower peak pressure, delivers maximum energy at the point of application on the skin and propagates outwards without a focal point [69,70,71] (Figure 1).

Thus, F-ESWT is considered to be more suitable for specific and deeper structures’ precise stimulation as the characteristics of the focal shockwave permit the standardized and reproducible stimulation of a small targeted area of tissue at a pre-defined depth in relation to the skin. This may help to circumvent the biases associated with palpation, such as the variable pressure intensity exerted by palpation and the stimulation of all the interposed tissues, which may contribute to the lack of sensitivity and specificity observed in numerous studies in this area [38,39,41,44]. Therefore, low to medium Energy Flux Density (EFD), used for myofascial ESWT along the anatomical course of nerve tracts, generates no local pain during application [39]. In contrast, MTrPs, which manifest as discrete structures, are identified through the patient’s pain response when stimulated. If the application is imprecise, the immediate surroundings of MTrPs are not painful, as determined by the corresponding feedback [38,39,40,41,42,43].

Previous studies using F-ESWT to target myofascial tissue in LBP document the muscles treated but did not report the exact identification of the MTrPs, which would be determined by the diagnostic criteria [44,66]. The objective of this study is to investigate whether F-ESWT is capable of reproducing the diagnostic criteria for MPS in a cohort of patients with LBP and to establish whether the use of focal shockwaves can be employed as a complementary tool in the clinical diagnosis of MPS. We hypothesize that F-ESWT, which delivers a reproducible, standardized mechanical stimulus in a non-invasive, targeted manner, could be employed as a diagnostic tool in order to obtain reproducible data.

## 2. Materials and Methods

The study was approved by the local ethics committee of the Universitätsklinikum of Munster, Germany (AZ 2022-303-f-S). Patients were enrolled between the 5th of July and the 31st of September 2022. Patients were required to sign the informed consent form before any procedure was performed. The study design and informed consent were in accordance with the Nuremberg Code and the latest revision of the Declaration of Helsinki and national laws and bylaws. This was a single-center, observational study designed to measure the effectiveness of a myofascial diagnostic assessment of the presence and location of MTrPs with F-ESWT in patients suffering from LBP, with an emphasis on the main diagnostic criteria of myofascial pain.

### 2.1. Study Design and Patient Selection

Patients were recruited through a newspaper advertisement with a diagnosis of chronic non-specific low back pain syndrome to our outpatient clinic. The diagnosis was provided by Hannes Müller-Ehrenberg and Federico Giordani, who are specialists in orthopedic and physical medicine and have more than six years of experience in the field of myofascial treatment. Demographic data, including age, gender, weight, height, body mass index, intensity and duration of the pain, was collected.

Inclusion criteria: (1) diagnosis of chronic non-specific LBP (more than 3 months duration with daily manifestations, (2) age between 20 and 70 years old.

Exclusion criteria: (1) neurological signs of spinal stenosis or loss of reflex or sensorimotor function (2) structural lesions such as spondylolysis or severe spondylolisthesis, secondary vertebral lesions, neoplastic origin (3) vascular etiology (i.e., abdominal aneurysms).

Step 1: patients were evaluated for the presence of myofascial trigger points by a physician with more than 6 years of experience in the field. Myofascial structures of the lower trunk, including the back and abdomen, as well as the gluteal region and thigh, were examined using manual palpation techniques for myofascial tissue and diagnostic criteria according to Travell/ Simons as presented in the introduction [6,20]. Myofascial structures of the lower trunk, including the back and abdomen, as well as the gluteal region and thigh, were examined by manual palpation. The localization of MTrPs is described using the established system of anatomical structures of muscles, as is common in most myofascial pain literature [6,20].

**Figure 2:**
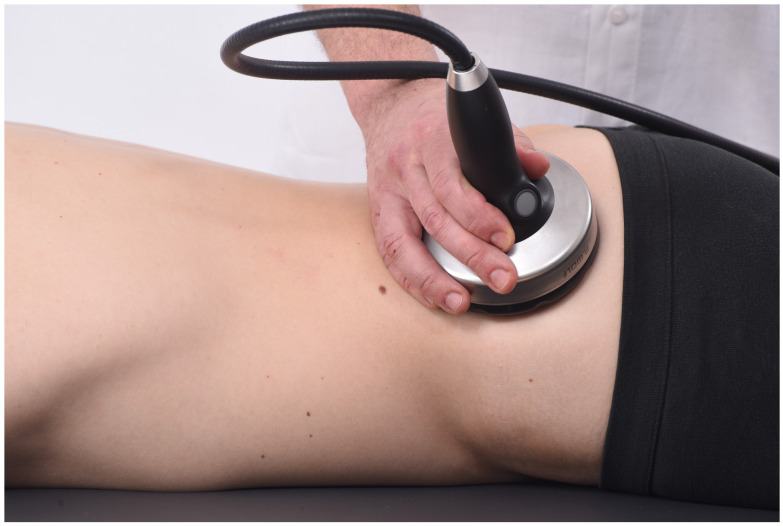
Example of the application of F-ESWT on the gluteal muscle.

Step 2: the selection of the MTrP for further investigation with focused shockwaves based on the clinical examination (choosing the most affected MTrPs) and the results of previous research on myofascial pain in LBP. The application of F-ESWT for MTrP examination was carried out by a physician with at least four years of experience in the use of F-ESWT (Figure 2). The examination was conducted using the Wolf PiezoWave2 FB10 G10 therapy source (Fa. Richard Wolf, Knittlingen, DE), with its precise focal zone of approximately 10mm in diameter (manufacturer’s specifications). In order to determine the required penetration depth, the previously performed palpation, which corresponded to the localization of the MTrPs, was taken into consideration. In addition, the appropriate penetration depth of the F-ESWT was determined, taking into account the individual’s constitution and the anatomical structures. Interchangeable gel pads used as spacers enabled the static focal area to be placed at the correct depth within the tissue (manufacturer’s specifications). The MTrPs in the deep lumbar spine and buttock area were reached with a penetration depth of 3–5 cm of focused shockwave. For the more superficial MTrPs, the focal zone was placed at a depth of 1–3 cm. The frequency used for the examination was set at 5 Hz, while the intensity, depending on the patient’s sensibility (subjective pain tolerance as stated vocally by the patient), was set between 0.06 and 0.30 mJ/mm^2^ Energy Flux Density (EFD).

**Figure 3:**
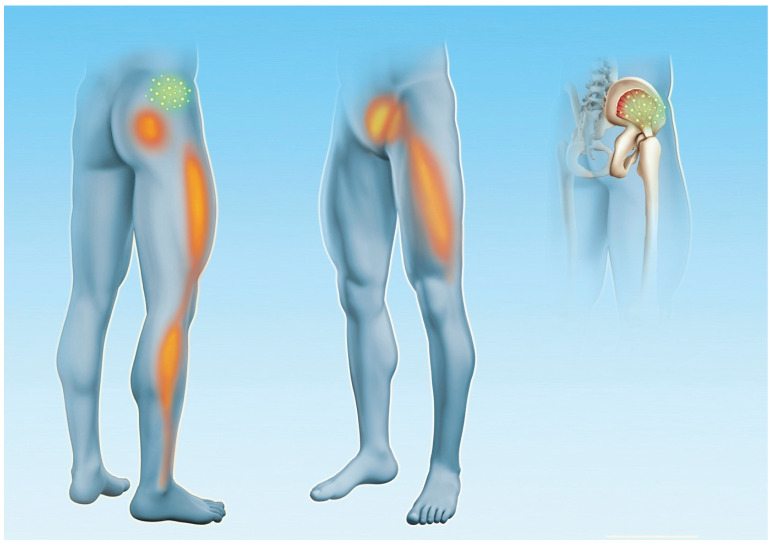
Referral of pain pattern of gluteus minimus muscle. The green area indicates the most common location of MTrPs, and the red/orange areas indicate the typical areas of the pain referral. Copyright by Hannes Müller-Ehrenberg.

During MTrP stimulation with F-ESWT to the muscles, patients were asked about referred pain: “Did you feel any pain locally and/ or in other areas?” and for recognition of pain: “Is this sensation and/ or pain familiar to you or is this pain part of your usual complaints?”. Possible answers were “yes” and “no” (Figure 3).

### 2.2. Statistics

A descriptive analysis was conducted using the Excel software for Windows version v28.0 (IBM Corp., Armonk, NY, USA) for data collection of the patient information and the characteristics of MTrPs of the investigated muscles. Age, body mass index, and duration of LBP were reported as mean values and standard deviations. Pain referral and pain recognition were recorded as binary variables (positive/negative) for every muscle tested. Variables were reported as the sum of positive registrations and as percentages of the total number of MTrPs stimulated for a given muscle. In patients with MTrPs on both sides, a positive referral of pain or recognition of the pain was recorded separately.

## 3. Results

Twenty-eight patients (F = 15; M = 13) matched the inclusion criteria and were included in the study protocol. Demographic and low back pain characteristics of the patients are listed in Table 1.

In step 1, we found several MTrPs in the myofascial structures of the lower trunk, including the back and abdomen, as well as in the gluteal region and lower limb. We selected a total of 115 MTrPs across all patients for ESWT stimulation.

The lower back and gluteal region showed the highest number of MTrPs (Table 2). After stimulation with ESWT, investigation of quadratus lumborum muscle (QL), gluteus medius muscle (GMed), and gluteus minimus muscle (GMin) reported the highest percentage of referred pain (96.15%, 95.65%, 92.86%, respectively) and recognition of the pain (84.62%, 86.96%, 85.71%, respectively). Erector spinae muscles (ES) (85.71%) and iliopsoas muscle (IP) (92.86%) showed a consistent percentage for the referred pain and a lower percentage for recognition of the pain (ES: 71.43% and IP 50%). Among the selected MTrP in the remaining muscle, biceps femoris muscle (BF), piriformis muscle (PM) and adductor muscle (Add) reported the highest referred pain (respectively 83.33%; 81.82%; 64.29%), although if a consistently lower percentage of “recognition of the pain” was found (Table 2). In one patient out of 28, neither recognition nor a referral of the pain was elicited.

**Figure 4:**
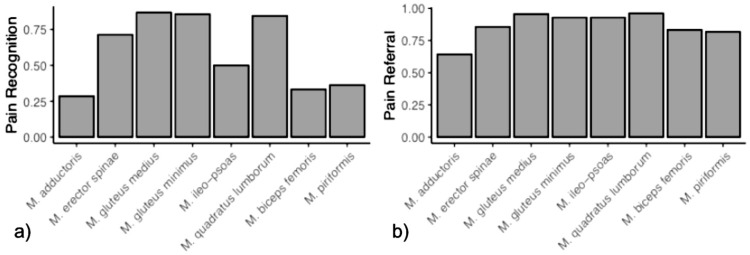
(**a**) pain recognition and (**b**) pain referral for each muscle by fESWT stimulation.

**Figure 5:**
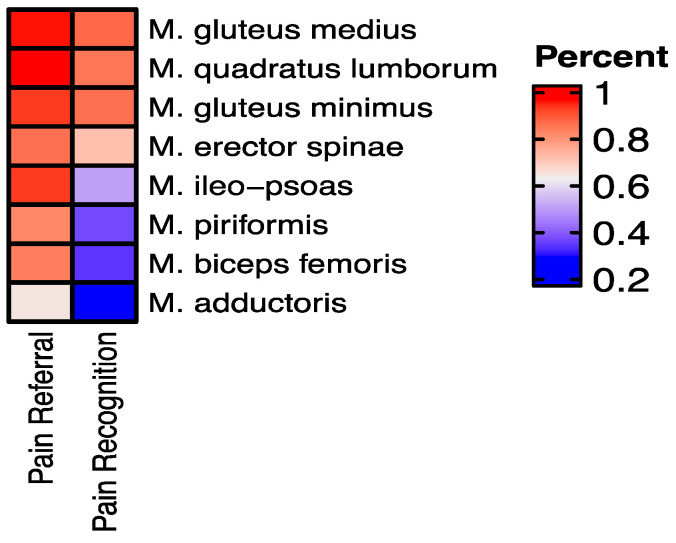
Heatmap of the frequency of pain recognition and pain referral by F-ESWT stimulation.

A graphic representation of the results is provided in Figure 4 and Figure 5.

## 4. Discussion

Studies in recent years indicate that muscles and fascia are more responsible for musculoskeletal pain than previously documented [13,29,53,54,57]. A substantial body of evidence indicates that myofascial syndrome and myofascial trigger points are significant contributors to LBP [12,13,22,24,29,35,54]. A precise and accurate diagnosis of myofascial pain syndrome is essential for the implementation of an appropriate clinical treatment plan and for the conduct of a comprehensive clinical study of myofascial pain. According to our study, F-ESWT is very frequently able to elicit the diagnostic criteria of recognition and referral of pain when directly applied to MTrPs in a cohort of patients affected by non-specific LBP. The findings lend support to the hypothesis that F-ESWT offers a uniform, non-invasive, targeted mechanical stimulus and could be utilized as a standard diagnostic instrument for low back pain, especially for MTrPs. To the best of our knowledge, no other studies have been published to date concerning the ability of F-ESWT to replicate the diagnostic criteria for MPS in LBP.

The role of MPS and MTrPs in the diagnosis of musculoskeletal complaints is not yet widely recognized. This is due to a lack of widespread knowledge of myofascial pain and the fact that the diagnostics used to date provide too few results that can be used in everyday clinical practice and scientific studies. This can be explained partly by the subjectivity of the examination and by the immaturity of previous instrumental examination techniques [15,16,19,33,34]. The majority of authors who have evaluated studies of palpation of MTrP in LBP have reached the conclusion that this examination method is not a valid diagnostic modality [14,15,16,19,21,29]. This is due to a number of factors, including the insufficiently qualified use of the diagnostic criteria and the unsatisfactory intra- and inter-rater reliability of palpation [14,29,30,31]. As regards the use of criteria, in their analysis of 198 studies examining myofascial pain and diagnostic criteria employed in physical therapy clinical trials, Li and colleagues [30] identified that 129 studies (65.1%) explicitly delineated the diagnostic criteria utilized for MTrPs in the main text. It appears that over one-third of the studies did not report a method for diagnosing MTrP or did not provide precise specifications. According to this review, the most frequently applied diagnostic criteria were “spot tenderness” (n = 125, 96.9%), “referred pain” (n = 95, 73.6%), “local twitch response” (n = 63, 48.8%), and pain recognition (n = 59, 45.7%). A study by Malanga et al. indicates that a significant number of studies restrict their analysis to the diagnostic criteria of “taut band and tenderness/tender spot,” which is employed in 63% of cases [29]. Additionally, the recognition of pain and referral of pain are identified in 53% and 44% of cases [29]. Thus, even if there is consensus that referral of pain and recognition are the most important criteria [13,14,18,22,23], many studies have not even mentioned it [29,30]. On the other hand, these criteria are probably not reported due to the difficulty of reproducing them manually [19,21,35]. Hua et al. examined the QL and GMed in 63 non-specific LBP patients using the palpation technique explicitly [21]. The combination of tenderness and referral was identified in QL in 10% of cases and in Gmed in 13% of cases. Conversely, the combination of tenderness and recognition in QL was observed in 36% of cases and in Gmed in 34% of cases [21]. In the study of Adelmanesh et al., a gluteal MTrP was defined via palpatory examination when the combination of a taut band, tenderness, and pain recognition was present in the superior outer quadrant of the gluteal muscles. The gluteal MTrP palpation was positive in 12 of 187 patients with non-radicular LBP (8.5%) and in 137 patients (74.1%) with radicular pain. The higher rate found in the radicular pain LBP group might be explained by the group selection, as preliminary evidence indicates that gluteal muscle palpation for tenderness may be a valid method for differentiating LBP with and without radiculopathy [24,72].

We observed in our study a high percentage of “recognition of pain” through F-ESWT stimulation of the MTrP of the QL (84%), GMed (86%), and GMin (85%). This is an important finding because “recognition” is considered by consensus to be the most important criterion in MPS [13,14,18,21,22,23]. In general, the reproduction of pain and the patient’s vocal feedback play a vital role in any clinical examination and are useful in understanding the patient’s complaints. This criterion not only helps to differentiate MTrPs from other sources of musculoskeletal pain but also reinforces the patient-centered nature of the diagnostic process, ensuring that diagnosis and treatment are tailored to the patient’s specific pain experience [6,13,14,24]. Recognition of the complaints through precise stimulation may indicate that the correspondingly stimulated structures are involved in the entire pain process or even dominantly cause it [8,14,24].

The typical “referral of pain” to the lower extremity, as described in textbooks, was detected in more than 90% of patients after stimulation of MTrP of the QL (96%), GMed (95%) and Gmin (92%) [6,20]. This is of great diagnostic importance as this symptomatology is often identical to radiculopathy, and these complaints can result in incorrect recommendations for surgery [7,60,61,73]. Indeed, it is evident from research findings that the deep fascia has a distinct pain propagation pattern, which differs from the overlying skin and underlying muscles [74,75]. Recent studies suggest that the innervation patterns of the deep fascia likely follow myotomes rather than dermatomes [76]. In fact, the anatomical organization of the deep fascia can result in significant irradiation of pain along the fascial reinforcements, which follow a distinctive pattern from that observed in the skin [76]. This distinction provides a rationale for the ‘anomalous’ pattern of pain irradiation that does not adhere to a specific dermatome, such as in the pseudo-radicular radiation in the lower extremity of LBP patients (see Figure 1). It was proposed that alterations in the dermatome result in the onset of clearly localized pain, while fasciatome-irradiated pain manifests in accordance with the organization of fascial anatomy [76]. Thus, stimulation of myofascial tissue by focused shockwaves could trigger the pain of myofascial origin and reproduce the “referral of pain” along the same pathways. This may prove useful in differentiating between radiculopathy and myofascial pain.

Furthermore, consistent with previous research [12,13,22,24,29,35,56], our study reports that the prevalence of MTrPs in the QL, GMed and Gmin is very high, and they play a major role in LBP. However, MTrPs were not only diagnosed in the obvious muscles involved in the complex pain pattern of LBP but were also frequently encountered in other muscles such as IP, Add, and ES. The identification of MTrP in structures distant from the site of pain suggests a wider involvement of the myofascial system at the origin of LBP.

Existing studies using F-ESWT and R-PP for LBP have shown satisfactory treatment results but a significant lack of diagnostic quality [44,51,55,63,64,66]. In most of these publications, the treated muscle is reported meaningfully, but the exact application to the MTrP is not described, and the diagnostic criteria are not even mentioned. This is unfortunate because the precision of the exact trigger point stimulation plays a major role in the success of the treatment of myofascial pain and in the reliable investigation of MPS through diagnostic criteria [10,14,40,42,43]. Furthermore, most of the studies have used R-PP, which results in maximum energy on the surface of the skin, thereby stimulating nociception of the skin rather than the underlying deeper muscles and fasciae. This may introduce a bias during the diagnostic and the targeting process on MTrP, which is similar to the palpation one. Indeed, the majority of MTrPs in LBP, as in QL and GMed, require deep stimulation (3–5cm of depth), which can be readily achieved through F-ESWT with the maximum energy (focus zone) directed towards the deeper layers, thus avoiding the superficial layers. Therefore, the nociception of myofascial tissue differs from the nociception of the skin, as described in the literature [54,76,77]. Skin nociception is described as being more localized, with no tendency towards referral of pain, and as a “stabbing, burning, or cutting” sensation, and on an affective level, as being tolerable. All these features are almost opposites to what is described for myofascial nociception [8,9,25].

The most significant clinical advantage of F-ESWT as a diagnostic instrument is its capacity to directly identify myofascial structures also in deeper tissue layers as the source of pain and to target them for specific therapeutic intervention. This may be a significant factor in the diagnostic process of LBP as it may assist in differentiating between the various causes of pain. As previously stated, there is a strong correlation between symptoms of radiculopathy and trigger points in the gluteal muscles. In these cases, diagnostic F-ESWT may play a significant role, as many of these patients are treated with surgery. In the literature, complaints that persist after lumbar spinal surgery are described under the term failed back surgical syndrome (FBSS) [78]. The incidence of FBSS is reported to range from 10% to 40% of cases [78,79]. It has been suggested that this high prevalence of postoperative complications is less attributable to incorrect surgical technique and more to incorrect surgical indication [80]. In light of the aforementioned considerations, the utilization of diagnostic F-ESWT in the context of LBP may facilitate a more precise surgical indication, thereby reducing the incidence of FBSS.

Thus, F-ESWT delivers a reproducible, standardized mechanical stimulus to the myofascial structures in a non-invasive, targeted manner and may be used as a standard tool for the diagnosis of LBP and particularly of MTrPs.

There are several limitations in our study. The study protocol was carried out as a proof of concept study in a single center and by a single investigator. This brings up a subjectivity issue and should be replicated in a larger-scale study. In addition, MTrPs were localized first by manual palpation and then further investigated by F-ESWT. This might introduce some bias or pose the question of whether manual palpation might not be sufficient. Scanning the entire body with F-ESWT is, of course, possible but very time-consuming. Thus, a pre-selection by manual palpation was chosen for practical reasons. Further research should investigate to which extent F-ESWT is superior to manual palpation in eliciting “referral” and “recognition” of pain, and other studies on the use of diagnostic ESWT are needed to confirm results in a wider population and other musculoskeletal diseases.

## 5. Conclusions

The present study has demonstrated that F-ESWT is capable of eliciting a remarkably high percentage of diagnostic criteria for pain recognition and referral in patients with LBP. Focused shock waves (F-ESWT) non-invasive nature, coupled with the potential for targeted application with standardized energy, makes it an attractive instrumental examination technique and may be used as a standard tool for the diagnosis of LBP and especially of MTrPs. We propose that the myofascial ESWT method should be learned both in terms of knowledge of myofascial pain and targeted application. It is a promising examination method that, in contrast to manual palpation, is not subjective and provides reproducible data that are directly related to the patient’s complaints. The utilization of diagnostic F-ESWT may facilitate the identification of more appropriate indications for a targeted treatment approach to low back pain (LBP). Further research is required to confirm the potential of F-ESWT as a diagnostic and treatment modality for LBP and to determine its comparative efficacy and therapeutic outcomes in relation to other treatment options.

## Figures and Tables

**Table 1:** Demographics and low back pain characteristics of the patients. SD: Standard Deviation; BMI: Body Mass Index; LBP: Low Back Pain; VAS: Visual Analog Scale.

n = 28	Age (years)	BMI (kg/m^2^)	Duration of LBP (months)	Rate of LBP (VAS)
Mean value (SD)	59.00 (7.60)	26.27 (4.17)	87.39 (98.19)	5.71 (1.86)

**Table 2:** Number of patients stimulated with F-ESWT on the myofascial trigger point for each muscle and corresponding provocation of referral of pain and recognition of the pain.

	N° of Patients with Significant MTrP by Manual Selection	Referred Pain During F-ESWT Stimulation		Recognition of Pain During F-ESWT Stimulation	
		N° of Patients	(%)	N° of Patients	(%)
M. quadratus lumborum	26	25	96.15	22	84.62
M. gluteus medius	23	22	95.65	20	86.96
M. gluteus minimus	14	13	92.86	12	85.71
M. ileo-psoas	14	13	92.86	7	50.00
M. erector spinae	7	6	85.71	5	71.43
M. biceps femoris	6	5	83.33	2	33.33
M. piriformis	11	9	81.82	4	36.36
M. adductoris	14	9	64.29	4	28.57

## Data Availability

Data are contained within the article.
